# Association Between Lipid Accumulation Product and Visceral Adiposity Index, as Markers of Visceral Adiposity and Metabolic Dysfunction, and Stroke: NHANES 1999–2018

**DOI:** 10.1155/ije/6820633

**Published:** 2026-10-17

**Authors:** Qian Zhou, Zhongchen Zhang

**Affiliations:** ^1^ Department of Critical Care Medicine The Fifth People’s Hospital of Jinan City Jinan 250022 China; ^2^ Department of Internal Medicine Shandong University Hospital Jinan 250100 China

**Keywords:** adipose tissue dysfunction, insulin resistance, lipid accumulation product, metabolic syndrome, NHANES, stroke, visceral adiposity, visceral adiposity index

## Abstract

**Aims:**

Lipid accumulation product (LAP) and visceral adiposity index (VAI) are surrogate markers of visceral adiposity‐related endocrine and metabolic dysfunction, including insulin resistance, rather than simple obesity indices. This study aimed to evaluate their associations with stroke prevalence using nationally representative data.

**Materials and Methods:**

This cross‐sectional study analyzed data from the National Health and Nutrition Examination Survey (NHANES) 1999–2018. Stroke status was determined through standardized questionnaires. LAP and VAI were calculated using established gender‐specific formulas. Analyses used the fasting subsample weights with strata and primary sampling units, and missing covariates were multiply imputed. Weighted logistic regression, restricted cubic spline (RCS), subgroup and incremental discrimination analyses were performed.

**Results:**

Among 21,146 participants, 721 reported a prior stroke (weighted prevalence 2.64%). Both indices were higher in stroke cases (median LAP 54 vs 42; VAI 1.72 vs 1.46, both *p* < 0.001). In the primary model (Model 3), each 1‐SD increment was associated with higher stroke odds for LAP (OR = 1.115, 95% CI 1.061–1.171, *p* < 0.001) and VAI (OR = 1.073, 95% CI 1.020–1.129, *p* = 0.007), and the highest quartile exceeded the lowest (LAP: OR = 1.557, 95% CI 1.117–2.170; VAI: OR = 1.381, 95% CI 1.026–1.858). Both were attenuated after additional adjustment for metabolic mediators (HbA1c, systolic blood pressure, eGFR and medication use), consistent with partial mediation. RCS analyses showed significant overall associations (LAP *p* < 0.001; VAI *p* = 0.041) without significant nonlinearity (*p* = 0.177 and 0.143). Only race/ethnicity modified the LAP‐stroke association (*p* for interaction = 0.009; non‐Hispanic Black OR = 1.427, 95% CI 1.231–1.654). Adding either index did not materially improve discrimination.

**Conclusions:**

Higher LAP and VAI values are associated with a greater prevalence of stroke after adjustment for demographic, socioeconomic, and lifestyle factors, and their attenuation after adjustment for metabolic variables is consistent with partial mediation through insulin resistance and its hemodynamic consequences. Both indices are inexpensive endocrine‐metabolic markers of visceral adipose tissue dysfunction and insulin resistance that warrant prospective evaluation.

## 1. Introduction

Stroke remains one of the leading causes of mortality and long‐term disability globally, imposing substantial societal and economic burdens [1]. According to the latest Global Burden of Disease Study 2021, stroke was responsible for approximately 7 million deaths and over 160 million disability‐adjusted life‐years lost worldwide, representing the second leading cause of death among noncommunicable diseases [1, 2]. The global stroke burden has increased dramatically over the past three decades, with incident strokes rising by 70% and stroke‐related deaths increasing by 44% between 1990 and 2021, with the majority of this burden concentrated in low‐ and middle‐income countries [3, 4]. Projections indicate that the global stroke burden will continue to escalate, particularly among younger populations and regions with lower sociodemographic development [5, 6]. Traditional cardiovascular risk factors including hypertension, diabetes mellitus, dyslipidaemia, and smoking have been well established in stroke pathogenesis. However, the role of adiposity—particularly its distribution and metabolic consequences—has gained increasing recognition as a critical determinant of cerebrovascular risk [7]. The rapid global increase in obesity prevalence has been identified as one of the fastest‐growing risk factors for stroke, with high body mass index (BMI) contributing significantly to the rising stroke burden worldwide [8].

Conventional anthropometric measures, particularly BMI and waist circumference, have substantial limitations in cardiovascular risk assessment as they fail to distinguish between lean mass and fat mass or provide information about regional fat distribution patterns crucial for metabolic health [9, 10]. Contemporary research has highlighted the critical importance of visceral adipose tissue dysfunction in cardiovascular disease pathogenesis, as visceral adipose tissue functions as a metabolically active endocrine organ that secretes numerous bioactive molecules affecting insulin sensitivity, inflammation, and vascular function [11, 12]. The inability of traditional anthropometric measures to accurately assess visceral adiposity has prompted the development of novel composite indices that integrate anthropometric and biochemical parameters to provide superior estimates of metabolic risk. The lipid accumulation product (LAP) and visceral adiposity index (VAI) represent two such innovative approaches to adiposity assessment. LAP, first proposed by Kahn in 2005, combines waist circumference with triglyceride levels to quantify lipid‐related adiposity accumulation, particularly reflecting visceral fat deposition and associated metabolic dysfunction [13]. VAI, developed by Amato and colleagues, incorporates waist circumference, BMI, triglycerides, and high‐density lipoprotein cholesterol in sex‐specific formulas to estimate visceral adipose tissue function and its associated cardiometabolic consequences [14].

Emerging evidence suggests that these composite indices demonstrate superior predictive value for cardiovascular outcomes compared to traditional anthropometric measures. Studies have consistently shown that LAP and VAI better identify individuals at high cardiovascular risk, even among those with normal BMI values [15, 16]. Furthermore, these indices have been associated with various metabolic abnormalities including insulin resistance, metabolic syndrome, and type 2 diabetes, suggesting their potential utility in comprehensive cardiovascular risk stratification [17, 18]. Recent studies have also demonstrated significant associations between LAP and VAI with coronary heart disease, myocardial infarction, and overall cardiovascular mortality in diverse populations [15, 19].

Despite growing evidence supporting the clinical utility of LAP and VAI in cardiovascular risk assessment, their specific relationships with stroke prevalence remain inadequately characterized. While several studies have examined associations between these indices and general cardiovascular outcomes [20], comprehensive analyses of their associations with stroke in large, nationally representative populations are limited. Understanding these relationships is particularly important given the distinct pathophysiological mechanisms underlying different cardiovascular endpoints and the potential for targeted prevention strategies based on adiposity distribution patterns. Therefore, this study aims to evaluate the associations of LAP and VAI with stroke prevalence using NHANES data from 1999 to 2018.

## 2. Methods

### 2.1. Study Design and Participants

This cross‐sectional study analyzed data from the National Health and Nutrition Examination Survey (NHANES) spanning from 1999 to 2018. NHANES is a nationally representative survey conducted by the Centers for Disease Control and Prevention that assesses the health and nutritional status of adults and children in the United States through standardized interviews, physical examinations, and laboratory tests. Participants were selected through three sequential exclusion steps: (1) participants aged < 20 years or with missing age were excluded; (2) participants lacking the fasting biochemical measures required for LAP and VAI calculation, or lacking a valid fasting subsample weight, were excluded; and (3) participants without stroke outcome data were excluded. Because missing covariate data were addressed by multiple imputation rather than case‐wise deletion, completeness of covariate information was not an eligibility criterion.

### 2.2. Assessment of Stroke

Stroke status was determined through standardized questionnaire responses during the NHANES household interview. Participants were asked [21]: “Has a doctor or other health professional ever told you that you had a stroke?” Responses to variable MCQ160F were recorded as yes or no, with affirmative responses classified as prevalent stroke cases; as the item records a lifetime history, the event may have preceded the examination at which LAP and VAI were measured. This self‐reported approach has been validated in previous epidemiological studies and demonstrates good concordance with medical records for major cardiovascular events.

### 2.3. Assessment of LAP and VAI

LAP and VAI were calculated using established formulas validated in previous research. LAP was computed using sex‐specific equations that incorporate waist circumference and fasting triglyceride levels. For males, LAP = (waist circumference‐65) × triglycerides; for females, LAP = (waist circumference‐58) × triglycerides, where waist circumference is measured in centimetres and triglycerides in mmol/L (concentrations reported in mg/dL were divided by 88.57) [13].

VAI calculation incorporated waist circumference, triglycerides, HDL cholesterol, and BMI using sex‐specific formulas. For males: VAI = (WC/(39.68 + 1.88×BMI)) × (TG/1.03) × (1.31/HDL); for females: VAI = (WC/(36.58 + 1.89×BMI)) × (TG/0.81) × (1.52/HDL) [14], where WC represents waist circumference in centimetres, BMI is BMI in kg/m^2^, TG is triglycerides in mmol/L, and HDL is HDL cholesterol in mmol/L.

Waist circumference measurements were obtained at the high point of the iliac crest during minimal respiration using standardized protocols. Fasting triglycerides and HDL cholesterol levels were measured from venous blood samples collected after at least 8 h of fasting, analyzed using enzymatic methods in certified laboratories following strict quality control procedures.

### 2.4. Measurement of Covariates

Demographic variables included age (continuous), sex (male/female), race/ethnicity (Mexican American, other Hispanic, non‐Hispanic White, non‐Hispanic Black, other races including multiracial), marital status (married or living with partner, single, divorced, or widowed), education level (less than high school, high school or equivalent, college, or above), and poverty‐income ratio (PIR), dichotomized at 1.30 into household income below or above the poverty line. Medication use was defined as self‐reported current use of antihypertensive, lipid‐lowering and antidiabetic agents. Diagnosed hypertension and diabetes were not entered as covariates because their principal determinants, systolic blood pressure, HbA1c, and drug treatment, were modeled directly. Health behaviors comprised smoking status (never smoke, smoking, smoked in the past) and alcohol consumption (never drinking, drinking). Anthropometric measurements included BMI calculated as weight in kilograms divided by height in meters squared and also grouped as normal (< 25), overweight (25–30) and obese (>= 30 kg/m2), and waist circumference measured at the high point of the iliac crest in accordance with the NHANES anthropometry procedures manual. Physical measurements including height, weight, and waist circumference were obtained using standardized protocols by trained technicians. Clinical laboratory measurements included glycated hemoglobin (HbA1c, %), systolic blood pressure (mmHg), serum creatinine (mg/dL), and estimated glomerular filtration rate (eGFR, mL/min/1.73 m^2^). Blood pressure was measured using a standardized protocol with a mercury sphygmomanometer. HbA1c was analyzed using high‐performance liquid chromatography. eGFR was calculated with the 2021 race‐free CKD‐EPI creatinine equation [22].

### 2.5. Statistical Analysis

All analyses were performed in R version 4.3.1 following the NHANES analytic guidelines [23]. Because LAP and VAI require fasting triglycerides, the fasting subsample weight was used and combined across the 10 cycles (WTSAF20 YR = WTSAF2YR/10, or WTSAF4YR x 2/10 for the 4‐year cycle); the design was declared with svydesign (ids = SDMVPSU, strata = SDMVSTRA), and all descriptive, regression, spline, subgroup and interaction analyses were design‐based. Continuous variables are reported as weighted medians with interquartile ranges and categorical variables as unweighted counts with weighted percentages, compared by design‐based Wilcoxon rank‐sum and Rao‐Scott chi‐square tests. Covariates with less than 20% missing values were multiply imputed by chained equations (mice, *m* = 5), and estimates were pooled by Rubin’s rules [24].

LAP and VAI were modeled per 1‐SD increment and as weighted quartiles with Q1 as reference, with *p* for trend from the quartile rank; per 10‐unit and raw scales are reported as sensitivity analyses. Four weighted logistic models (svyglm) were fitted: Model 1 unadjusted; Model 2 adjusted for age, sex, and race/ethnicity; Model 3 (primary) additionally for marital status, education level, PIR, smoking status, and alcohol consumption; and Model 4 (exploratory) additionally for HbA1c, systolic blood pressure, eGFR, and antihypertensive, lipid‐lowering, and antidiabetic medication use. Covariates in Models 2 and 3 were prespecified confounders, whereas the clinical variables of Model 4 are potential mediators of the adiposity to stroke pathway. Both indices were mean‐centered before their product term was fitted and variance inflation factors were computed, with VIF > 5 indicating serious collinearity. Restricted cubic splines with three knots at the weighted 10th, 50^th^, and 90th percentiles were fitted on Model 3 with the 10th percentile as reference, tested by Wald tests for overall association and nonlinearity, truncated at the 1st to 99th percentiles and refitted after natural logarithmic transformation. Subgroup analyses included exposure‐by‐stratum product terms to obtain *p* for interaction and were exploratory. Incremental value over Model 3 was assessed by AUC (DeLong test), continuous NRI and IDI, and complete‐case and pooled imputed estimates were compared. Significance was set at *p* < 0.05.

## 3. Results

### 3.1. Basic Characteristics

The initial NHANES 1999–2018 dataset comprised 101,316 participants. After exclusion of those aged < 20 years or with missing age (*n* = 46,235), those lacking the data required for LAP and VAI calculation (*n* = 33,914), and those without stroke data (*n* = 21), 21,146 participants remained, of whom 721 reported a stroke (weighted prevalence 2.64%; Figure 1). Weighted baseline characteristics by stroke status are shown in Table 1, in which counts are unweighted and percentages and medians are weighted.

**FIGURE 1:**
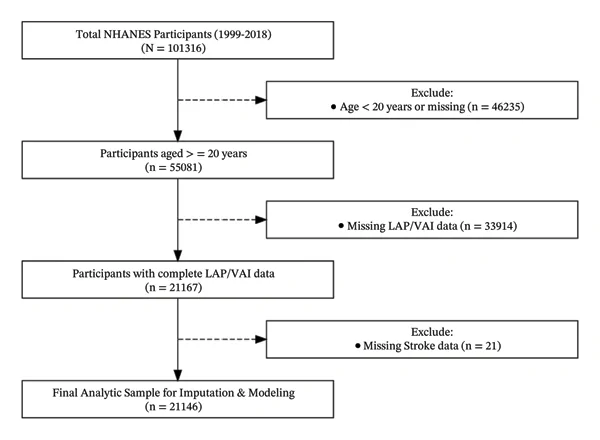
Three‐step flow chart for inclusion and exclusion of study participants.

**TABLE 1:** Weighted baseline characteristics of participants by stroke status.

Characteristic	Overall (*n* = 21,146)	Stroke: No (*n* = 20,425)	Stroke: Yes (*n* = 721)	*p*
Age (years)	46 (33, 59)	45 (32, 58)	66 (54, 76)	< 0.001
Sex				0.004
Male	10,193 (48%)	9846 (49%)	347 (42%)	
Female	10,953 (52%)	10,579 (51%)	374 (58%)	
Race/Ethnicity				< 0.001
Mexican American	3843 (8.3%)	3761 (8.4%)	82 (4.5%)	
Other Hispanic	1817 (5.6%)	1777 (5.6%)	40 (3.1%)	
Non‐Hispanic White	9469 (68%)	9099 (68%)	370 (70%)	
Non‐Hispanic Black	4057 (11%)	3872 (11%)	185 (15%)	
Other Race	1960 (6.9%)	1916 (6.8%)	44 (7.8%)	
Marital status				< 0.001
Married or living with partner	13,153 (65%)	12,750 (65%)	403 (63%)	
Single	3541 (17%)	3491 (18%)	50 (5.1%)	
Divorced or widowed	4452 (18%)	4184 (17%)	268 (32%)	
Education level				< 0.001
Less than high school	5598 (17%)	5327 (17%)	271 (29%)	
High school or equivalent	4845 (24%)	4661 (24%)	184 (30%)	
College or above	10,703 (59%)	10,437 (59%)	266 (41%)	
Poverty‐Income Ratio				< 0.001
Below poverty line	6418 (21%)	6136 (21%)	282 (32%)	
Above poverty line	14,728 (79%)	14,289 (79%)	439 (68%)	
Smoking status				< 0.001
Smoked in the past	4290 (21%)	4117 (21%)	173 (26%)	
Smoking	5350 (25%)	5074 (25%)	276 (37%)	
Never smoke	11,506 (53%)	11,234 (54%)	272 (37%)	
Alcohol consumption				0.002
Never drinking	5853 (23%)	5616 (23%)	237 (30%)	
Drinking	15,293 (77%)	14,809 (77%)	484 (70%)	
Body mass index (kg/m2)	28 (24, 32)	28 (24, 32)	29 (25, 34)	< 0.001
Waist circumference (cm)	97 (87, 108)	97 (87, 108)	103 (92, 114)	< 0.001
Systolic blood pressure (mmHg)	119 (110, 130)	119 (109, 130)	128 (115, 144)	< 0.001
Diastolic blood pressure (mmHg)	71 (63, 77)	71 (64, 77)	69 (60, 77)	0.003
HbA1c (%)	5.40 (5.10, 5.70)	5.40 (5.10, 5.70)	5.70 (5.30, 6.10)	< 0.001
eGFR (mL/min/1.73m2)	100 (85, 113)	100 (86, 113)	79 (60, 96)	< 0.001
Lipid accumulation product	42 (23, 75)	42 (23, 74)	54 (32, 87)	< 0.001
Visceral adiposity index	1.47 (0.89, 2.49)	1.46 (0.89, 2.48)	1.72 (1.06, 2.89)	< 0.001
Antihypertensive medication				< 0.001
No	14,080 (71%)	13,932 (72%)	148 (24%)	
Yes	7066 (29%)	6493 (28%)	573 (76%)	
Lipid‐lowering medication				< 0.001
No	17,398 (84%)	17,040 (85%)	358 (47%)	
Yes	3748 (16%)	3385 (15%)	363 (53%)	

*Note:* Data are median (Q1, Q3) or unweighted *n* (weighted %). Weighted population sizes: 207,919,986 overall, 202,428,914 without stroke and 5,491,071 with stroke. *p* values from design‐based Wilcoxon rank‐sum tests and Rao‐Scott chi‐square tests, using the combined fasting subsample weight and imputed dataset 1.

Participants with stroke were older than those without stroke (median 66 [54, 76] vs 45 [32, 58] years, *p* < 0.001) and included a higher proportion of women (58% vs 51%, *p* = 0.004). Non‐Hispanic Black participants accounted for a larger share of the stroke group (15% vs 11%) and Mexican American participants for a smaller share (4.5% vs 8.4%, *p* < 0.001). Educational attainment was lower among participants with stroke (29% vs 17% with less than high school education, *p* < 0.001), a higher proportion were divorced or widowed (32% vs 17%, *p* < 0.001), and a higher proportion had household income below the poverty line (32% vs 21%, *p* < 0.001). Current smoking (37% vs 25%, *p* < 0.001) and never drinking (30% vs 23%, *p* = 0.002) were also more frequent in the stroke group.

Participants with stroke had higher BMI (29 [25, 26] vs 28 [24, 27] kg/m^2^, *p* < 0.001) and waist circumference (103 [92, 114] vs 97 [87, 108] cm, *p* < 0.001). They also had higher HbA1c (5.70 [5.30, 6.10] vs 5.40 [5.10, 5.70] %, *p* < 0.001) and systolic blood pressure (128 [115, 144] vs 119 [109, 130] mmHg, *p* < 0.001), lower eGFR (79 [60, 96] vs 100 [86, 113] mL/min/1.73m2, *p* < 0.001), and more frequent use of antihypertensive (76% vs 28%) and lipid‐lowering (53% vs 15%) medication (both *p* < 0.001).

Both indices were higher in participants with stroke: median LAP was 54 (32, 87) versus 42 (23, 74) and median VAI was 1.72 (1.06, 2.89) versus 1.46 (0.89, 2.48) (both *p* < 0.001). Baseline characteristics before and after multiple imputation were closely comparable (Table S1), and the weighted quartile cutpoints of both indices are given in Table S2.

### 3.2. Association Between LAP, VAI, and Stroke

Weighted logistic regression results for LAP and VAI are shown in Table 2. In the unadjusted Model 1, each 1‐SD increment was associated with higher stroke odds for LAP (OR 1.123, 95% CI 1.064–1.184, *p* < 0.001) and for VAI (OR 1.084, 95% CI 1.037–1.133, *p* < 0.001).

**TABLE 2:** Weighted logistic regression analysis of LAP and VAI with prevalent stroke.

Exposure	Model 1 OR (95% CI)	*p*	Model 2 OR (95% CI)	*p*	Model 3 OR (95% CI)	*p*	Model 4 OR (95% CI)	*p*
LAP (per 1‐SD)	1.123 (1.064–1.184)	< 0.001	1.129 (1.070–1.191)	< 0.001	1.115 (1.061–1.171)	< 0.001	1.038 (0.954–1.129)	0.382
LAP Q1	1.000 (Reference)	—	1.000 (Reference)	—	1.000 (Reference)	—	1.000 (Reference)	—
LAP Q2	1.958 (1.408–2.723)	< 0.001	1.290 (0.927–1.794)	0.130	1.291 (0.930–1.792)	0.126	1.120 (0.802–1.564)	0.504
LAP Q3	2.141 (1.525–3.005)	< 0.001	1.297 (0.913–1.841)	0.145	1.249 (0.888–1.757)	0.200	0.983 (0.689–1.403)	0.925
LAP Q4	2.615 (1.882–3.633)	< 0.001	1.723 (1.237–2.401)	0.001	1.557 (1.117–2.170)	0.009	1.015 (0.717–1.437)	0.933
LAP *p* for trend	—	< 0.001	—	0.002	—	0.014	—	0.760
VAI (per 1‐SD)	1.084 (1.037–1.133)	< 0.001	1.101 (1.042–1.163)	< 0.001	1.073 (1.020–1.129)	0.007	1.023 (0.939–1.116)	0.597
VAI Q1	1.000 (Reference)	—	1.000 (Reference)	—	1.000 (Reference)	—	1.000 (Reference)	—
VAI Q2	1.328 (0.943–1.871)	0.104	1.211 (0.857–1.712)	0.275	1.131 (0.797–1.605)	0.488	0.992 (0.701–1.404)	0.962
VAI Q3	1.547 (1.150–2.080)	0.004	1.292 (0.946–1.764)	0.106	1.159 (0.849–1.583)	0.351	0.941 (0.688–1.288)	0.704
VAI Q4	1.917 (1.450–2.534)	< 0.001	1.663 (1.241–2.227)	< 0.001	1.381 (1.026–1.858)	0.033	1.001 (0.732–1.368)	0.997
VAI *p* for trend	—	< 0.001	—	< 0.001	—	0.024	—	0.958

*Note:* Model 1: unadjusted. Model 2: adjusted for age, sex and race/ethnicity. Model 3 (primary model): Model 2 plus marital status, education level, poverty‐income ratio, smoking status and alcohol consumption. Model 4 (exploratory model): Model 3 plus HbA1c, systolic blood pressure, eGFR, antihypertensive, lipid‐lowering, and antidiabetic medication use. All models were fitted with svyglm using the combined fasting subsample weight and pooled across five imputed datasets.

In the unadjusted quartile analysis, participants in the highest LAP quartile had higher odds of stroke than those in the lowest quartile (OR 2.615, 95% CI 1.882–3.633, *p* < 0.001), as did participants in the highest VAI quartile (OR 1.917, 95% CI 1.450–2.534, *p* < 0.001), with significant trends across quartiles for both indices (both *p* for trend < 0.001).

After adjustment for age, sex, and race/ethnicity in Model 2, the associations were attenuated but remained significant, both per 1‐SD (LAP: OR 1.129, 95% CI 1.070–1.191; VAI: OR 1.101, 95% CI 1.042–1.163, both *p* < 0.001) and for the highest versus the lowest quartile (LAP: OR 1.723, 95% CI 1.237–2.401, *p* < 0.001; VAI: OR 1.663, 95% CI 1.241–2.227, *p* < 0.001).

In the primary Model 3, which additionally adjusted for marital status, education level, PIR, smoking status, and alcohol consumption, each 1‐SD increment remained associated with stroke for LAP (OR 1.115, 95% CI 1.061–1.171, *p* < 0.001) and VAI (OR 1.073, 95% CI 1.020–1.129, *p* = 0.007). The highest quartile retained higher odds than the lowest for LAP (OR 1.557, 95% CI 1.117–2.170, *P* = 0.009; *p* for trend = 0.014) and VAI (OR 1.381, 95% CI 1.026–1.858, *p* = 0.033; *p* for trend = 0.024), whereas the intermediate quartiles did not differ significantly from Q1, so a strictly monotonic gradient was not demonstrated.

In the exploratory Model 4, which further adjusted for HbA1c, systolic blood pressure, eGFR, and medication use, both associations were attenuated to the null (LAP per 1‐SD: OR 1.038, 95% CI 0.954–1.129, *p* = 0.382; VAI per 1‐SD: OR 1.023, 95% CI 0.939–1.116, *p* = 0.597), and no quartile contrast or trend test remained significant (all *p* > 0.05).

Per 10‐unit and raw continuous scales gave consistent results (Table S4), and complete‐case estimates were almost identical to the pooled imputed estimates (Table S5).

Mean‐centered LAP and VAI remained strongly collinear (VIF 14.273 and 12.385; Table S3) and the interaction odds ratio was 1.000 (95% CI 1.000–1.000) in every model (Model 3 *p* = 0.064; Table S4); such estimates are not interpretable, and no inference about independent or additive pathways was drawn.

### 3.3. Subgroup Analyses

For LAP per 1‐SD increment, estimates were significant in participants aged ≤ 60 years (OR 1.127, 95% CI 1.064–1.194) and in obese participants (OR 1.098, 95% CI 1.035–1.165) but not in older, normal‐weight, or overweight strata, and none of these contrasts was supported by a significant interaction (*p* for interaction 0.621 for age, 0.359 for BMI, and 0.671 for sex). The only significant effect modification was by race/ethnicity (*p* for interaction = 0.009): the association was stronger in non‐Hispanic Black participants (OR 1.427, 95% CI 1.231–1.654) than in non‐Hispanic White participants (OR 1.099, 95% CI 1.023–1.182) and null in Mexican American participants (OR 0.997, 95% CI 0.772–1.289) (Figure 2).

**FIGURE 2:**
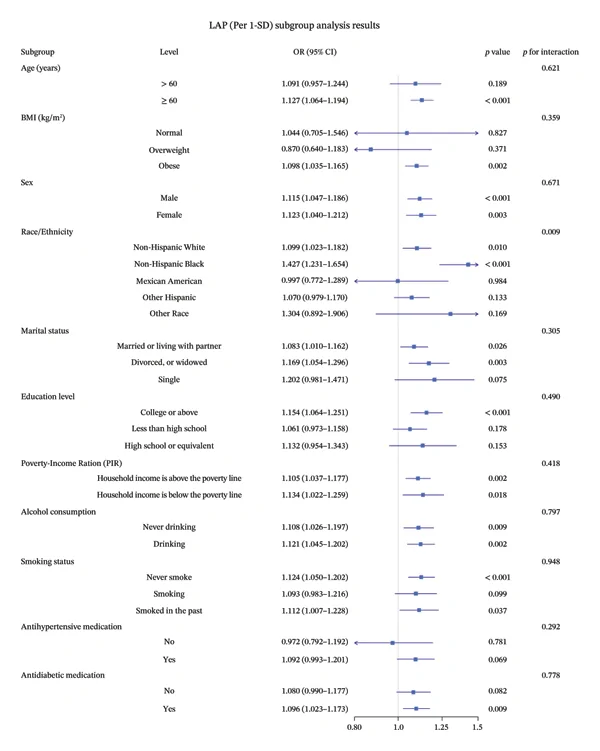
Subgroup analysis of the association between LAP (per 1‐SD increase) and stroke, with *p* for interaction for each stratifying variable.

For VAI per 1‐SD increment, no interaction was significant (all *p* > = 0.064), including race/ethnicity (*p* = 0.064) despite the larger estimate in non‐Hispanic Black participants (OR 1.537, 95% CI 1.227–1.924). Estimates were similar in men (OR 1.086, 95% CI 1.000–1.179) and women (OR 1.069, 95% CI 1.001–1.142) (Figure 3).

**FIGURE 3:**
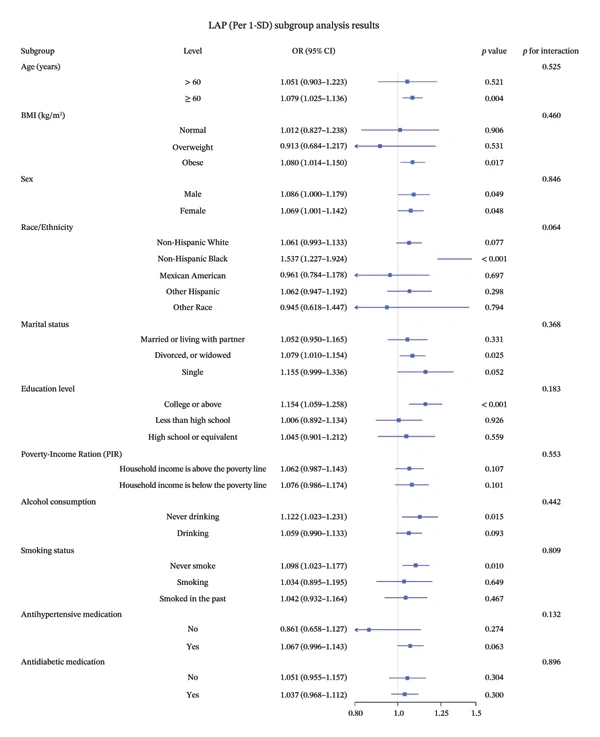
Subgroup analysis of the association between VAI (per 1‐SD increase) and stroke, with *p* for interaction for each stratifying variable.

Apart from race/ethnicity for LAP, the subgroup results are exploratory: significance in one stratum together with nonsignificance in another does not establish effect modification, and multiplicity remains a concern.

### 3.4. RCS Analyses

Spline models fitted on Model 3 showed significant overall associations for LAP (*p* < 0.001) and VAI (*p* = 0.041), with knots at the weighted 10th, 50th, and 90th percentiles (LAP: 12.79, 42.27, and 118.92; VAI: 0.59, 1.47, and 4.03) and the 10th percentile as reference. Nonlinearity was not significant (LAP *p* = 0.177; VAI *p* = 0.143), so the flattening of both curves at high values is compatible with a monotonic association rather than a threshold (Figure 4). Results were unchanged after logarithmic transformation (*p* for overall 0.006 for ln‐LAP and 0.070 for ln‐VAI; *p* for nonlinearity 0.568 and 0.826; Figure S1).

**FIGURE 4:**
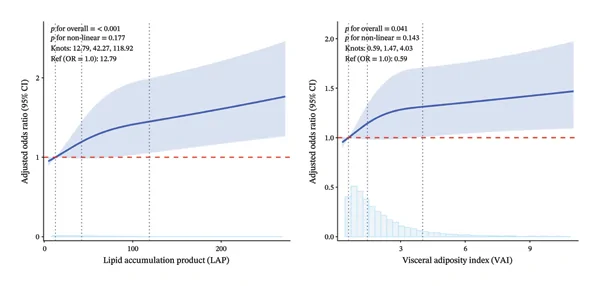
Weighted restricted cubic spline curves for LAP (left) and VAI (right) with stroke, fitted on Model 3. Knots were placed at the weighted 10th, 50th and 90th percentiles (LAP: 12.79, 42.27 and 118.92; VAI: 0.59, 1.47 and 4.03), the 10th percentile was the reference (OR = 1.000), and the plotted range is truncated at the 1st to 99th percentiles.

### 3.5. Incremental Predictive Value

Adding either index to the primary model changed discrimination only marginally (Table 3): AUC was 0.801 (95% CI 0.787–0.814) for Model 3, 0.802 with LAP (DeLong *p* = 0.033), and 0.801 with VAI (*p* = 0.216). The continuous NRI was significant for both indices (LAP 0.211; VAI 0.186, both *p* < 0.001), whereas the IDI was essentially zero (*p* = 0.101 and 0.233), indicating no material improvement in model performance.

**TABLE 3:** Incremental predictive value of LAP and VAI over the primary model.

Model	AUC (95% CI)	*p* for delta AUC	Continuous NRI (95% CI)	*p*	IDI (95% CI)	*p*
Base model (Model 3)	0.801 (0.787–0.814)	—	—	—	—	—
Base model + LAP	0.802 (0.789–0.816)	0.033	0.211 (0.139–0.284)	< 0.001	0.001 (−0.000–0.001)	0.101
Base model + VAI	0.801 (0.788–0.815)	0.216	0.186 (0.115–0.257)	< 0.001	0.000 (−0.000–0.001)	0.233

*Note:* AUC, area under the receiver operating characteristic curve. Differences in AUC were tested with the DeLong test.

Abbreviations: IDI = integrated discrimination improvement, NRI = net reclassification improvement.

## 4. Discussion

The present study revealed potential associations between LAP, VAI, and stroke prevalence among US adults, with these novel adiposity indices showing meaningful relationships with cerebrovascular outcomes. These findings extend a previous NHANES analysis of VAI and combined cardiovascular and cerebrovascular endpoints in the same cycles: stroke was analyzed as a discrete outcome, LAP was added on a per‐SD scale, fasting weights and multiple imputation were applied, and interaction and incremental discrimination were tested. From an endocrine and metabolic standpoint, both indices act as proxies for insulin resistance, metabolic syndrome, and adipose tissue dysfunction rather than for fat mass, and both discriminate prediabetes and diabetes in the general population [25]; stroke can therefore be viewed as one consequence of visceral adiposity‐related metabolic dysfunction.

Both LAP and VAI were associated with stroke prevalence in the primary model, with LAP showing the slightly larger per‐SD estimate. The highest quartile of either index carried higher odds than the lowest, but the intermediate quartiles did not differ from the reference, so a strictly monotonic gradient cannot be claimed. Notably, both associations were attenuated to the null once HbA1c, systolic blood pressure, renal function, and medication use were added, suggesting that a substantial part of the association is shared with, or mediated by, glycemic and hemodynamic dysregulation [14]. Because these variables plausibly lie on the pathway between visceral adiposity and cerebrovascular disease, Model 4 is best read as a conservative sensitivity analysis, and the attenuation indicates a weaker signal independent of metabolic mediators. Both indices capture the metabolic derangements associated with visceral adiposity that have been described in recent prospective cohort studies [28, 29]. Comparing our findings with prospective studies, a large‐scale Chinese cohort study reported that VAI was independently associated with incident stroke with a hazard ratio (HR) of 1.45 (95% CI: 1.15–1.75) for the highest quartile versus the lowest quartile in fully adjusted models [30]. Similarly, another Chinese cohort study demonstrated an odds ratio of 2.33 (95% CI: 1.67–3.28) for new‐onset stroke in the highest CVAI quartile [31]. The effect sizes in our cross‐sectional analysis (OR 1.381, 95% CI 1.026–1.858 for VAI Q4 vs Q1 in Model 3) are smaller than in these prospective studies. This discrepancy likely reflects fundamental differences in study design: cross‐sectional studies assess prevalent cases and capture only associations at a single time point, while prospective cohort studies establish temporal relationships and measure incident events, potentially yielding stronger effect estimates. Additionally, survivor bias in cross‐sectional studies may attenuate associations, as individuals with the most severe manifestations of visceral adiposity dysfunction may have experienced fatal strokes prior to study enrollment. An earlier NHANES analysis reported nonlinear associations of VAI with cardiovascular and cerebrovascular disease, which we could not confirm for prevalent stroke after weighting and imputation [29].

The RCS analyses showed significant overall associations for both indices but no significant departure from linearity (LAP *p* for nonlinearity = 0.177; VAI *p* = 0.143). Stroke odds rose progressively across the observed range, and the flattening of both curves at high values probably reflects sparse data at the extremes. This differs from earlier NHANES‐based reports of nonlinear associations between VAI and cardiovascular outcomes [29], and a threshold value for clinical decision‐making cannot be derived from the present data. The LAP‐VAI product term was uninterpretable and the indices were severely collinear, so no conclusion about independent or additive pathways can be drawn; conceptually, they capture complementary aspects of visceral adipose tissue dysfunction, LAP primarily reflecting lipid overaccumulation and VAI more broadly integrating adipose tissue function.

The mechanistic basis for the observed associations involves multiple interconnected pathways linking visceral adipose tissue dysfunction to cerebrovascular pathology. Visceral adipose tissue functions as a metabolically active endocrine organ, secreting numerous bioactive molecules that influence systemic inflammation, insulin sensitivity, and vascular function [32, 33]. Recent research has elucidated that dysfunctional visceral adipocytes secrete pro‐inflammatory cytokines including tumor necrosis factor‐α, interleukin‐6, and monocyte chemoattractant protein‐1, creating a state of chronic low‐grade systemic inflammation that promotes atherosclerosis and vascular dysfunction [27, 34].Contemporary evidence suggests that visceral adiposity contributes to stroke risk through several key mechanisms. First, visceral adipose tissue dysfunction promotes insulin resistance and metabolic syndrome, creating a pro‐thrombotic milieu characterized by elevated plasminogen activator inhibitor‐1 and fibrinogen levels [26]. Second, dysfunctional adipocytes release increased amounts of free fatty acids, leading to lipotoxicity in vascular endothelial cells and acceleration of atherosclerotic plaque formation [35, 36]. Third, visceral adiposity is associated with activation of the renin–angiotensin–aldosterone system, contributing to hypertension and vascular remodeling [37]. Recent mechanistic studies have also highlighted the role of adipokines in stroke pathogenesis. A systematic review and meta‐analysis demonstrated that adipokine dysregulation, particularly decreased adiponectin and increased leptin levels, was significantly associated with stroke risk independent of traditional risk factors [38]. The visceral adiposity inflammatory index, which combines VAI with high‐sensitivity C‐reactive protein, has shown superior predictive accuracy for stroke risk compared to either measure alone, emphasizing the critical role of inflammation in mediating the adiposity–stroke relationship [39]. From an endocrine perspective, both indices are markers of insulin resistance and the metabolic syndrome rather than of fat mass, consistent with the attenuation of our estimates after adjustment for HbA1c. Dysfunctional visceral adipocytes shift adipokine secretion towards leptin and inflammatory cytokines and away from adiponectin, which impairs insulin signaling, raises circulating triglycerides, and lowers HDL cholesterol [40]; because these are the components of LAP and VAI, both indices can be regarded as readouts of the endocrine activity of visceral fat. Visceral adiposity, dyslipidaemia, and inflammation also promote atrial remodeling and atrial fibrillation, a major substrate of cardioembolic stroke [41]. Atrial fibrillation was not recorded in all cycles and could not be adjusted for, so part of the association may operate through arrhythmic mechanisms.

The current findings are consistent with evidence that composite adiposity indices are associated with cardiovascular outcomes, although we did not compare them directly with BMI or waist circumference. A prospective study utilizing data from the China Health and Retirement Longitudinal Study found that higher VAI levels were associated with increased stroke incidence over 9 years of follow‐up, with similar effect sizes to those observed in the present analysis [31]. Analyses in other populations suggest that LAP and VAI may identify individuals at high cardiovascular risk who are missed by BMI and waist circumference [15, 16]. However, the magnitude of associations observed in the current study was somewhat attenuated compared to some previous reports, likely reflecting the comprehensive adjustment for confounding variables employed in the analysis. An Iranian cohort study of VAI and the atherogenic index of plasma reported stronger estimates for the highest versus the lowest category than we observed, a difference attributable to its incident design and population characteristics [42].

The subgroup analyses were exploratory, and only race/ethnicity modified the LAP‐stroke association. Differences between strata in the statistical significance of individual estimates therefore do not indicate sex‐ or age‐specific effects. Estimates in women and men were closely similar and the sex interaction was not significant (*p* = 0.671 for LAP, and *p* = 0.846 for VAI), although sex differences in visceral adipose tissue distribution and function are well described [43, 44]. Women tend to accumulate more subcutaneous adipose tissue during premenopausal years due to estrogen’s protective effects, but after menopause, visceral fat accumulation accelerates dramatically. This postmenopausal shift in fat distribution is accompanied by greater metabolic dysfunction per unit of visceral fat in women compared to men. Furthermore, visceral adipose tissue in women exhibits higher inflammatory cytokine secretion and reduced adiponectin production, potentially amplifying the cardiometabolic risk associated with visceral adiposity. These mechanisms remain biologically plausible but are not supported as effect modifiers by the present data. The stronger LAP–stroke association among non‐Hispanic Black participants, the only finding supported by a significant interaction (*p* = 0.009), may reflect the higher burden of cardiometabolic risk factors and visceral adiposity in this population. No interaction with education or household income was detected, so the corresponding gradients should not be over‐interpreted. Recent population‐based studies have demonstrated that the relationship between adiposity indices and cardiovascular outcomes is modified by socioeconomic factors, with stronger associations observed in higher education groups, possibly due to better recognition and management of cardiovascular risk factors [45].

The findings have several important implications for clinical practice and public health policy. First, although both indices are associated with prevalent stroke, adding them to the primary model did not materially improve discrimination in this cross‐sectional setting, so risk stratification cannot be claimed on the basis of these data. Their value lies rather in being inexpensive descriptive markers of adipose tissue dysfunction and insulin resistance, computable from waist circumference, BMI, triglycerides, and HDL cholesterol [46]. Second, because the design is cross‐sectional and the temporal order of exposure and outcome is unknown, these data cannot show that lowering visceral adiposity reduces stroke occurrence. No departure from linearity was detected, so no cut‐point for intervention can be recommended. Observational studies indicate that visceral adiposity tracks with the progression of subclinical cardiovascular disease and with type 2 diabetes, but interventional evidence that modifying these indices alters stroke occurrence is lacking [47, 48].

Several limitations should be acknowledged. The design precludes causal inference: stroke was a lifetime history, whereas LAP and VAI were measured at the examination, so in many participants, the event preceded the measurement, and post‐stroke weight loss, diet change, and drug treatment may have altered the components of both indices. Reverse causation is therefore a concern, and all estimates describe prevalent stroke. Stroke was ascertained from a single self‐reported item and is subject to recall and misclassification bias. NHANES does not distinguish ischemic from haemorrhagic stroke, and because lipids and adiposity have partly opposing roles in the two subtypes, analyzing them jointly dilutes the mechanisms involved and may bias estimates towards the null. Low LDL cholesterol, for instance, is associated with a higher risk of intracerebral hemorrhage whereas higher concentrations increase ischemic risk [49]. Only stroke survivors could take part, so fatal and most severe cases are absent, which probably attenuates the effect sizes relative to prospective cohorts. Residual confounding from physical activity, diet, and atrial fibrillation cannot be excluded, and participants without fasting measurements remained excluded despite imputation. Future research should focus on prospective validation of these adiposity indices in diverse populations, investigation of optimal cut‐points for clinical decision‐making, and evaluation of whether interventions targeting visceral adiposity translate to reduced stroke incidence. The development of refined indices incorporating additional biomarkers of adipose tissue dysfunction, such as adipokines and inflammatory markers, represents a promising avenue for enhanced risk prediction.

## 5. Conclusion

This weighted analysis shows that higher LAP and VAI are associated with a greater prevalence of stroke among US adults after adjustment for demographic, socioeconomic, and lifestyle factors, and the attenuation observed after further adjustment for glycemic, hemodynamic, renal, and treatment variables is consistent with partial mediation through these metabolic pathways. No departure from linearity was detected for either index, and effect modification was confined to race/ethnicity for LAP. These findings are consistent with a contribution of visceral adiposity‐related metabolic dysfunction to stroke, and LAP and VAI are therefore inexpensive, routinely computable endocrine‐metabolic markers of adipose tissue dysfunction and insulin resistance that merit evaluation in prospective studies.

## Author Contributions

Qian Zhou and Zhongchen Zhang carried out the studies, participated in collecting data, and drafted the manuscript. Qian Zhou performed the statistical analysis and participated in its design. Qian Zhou and Zhongchen Zhang helped to draft the manuscript.

## Funding

This research did not receive any specific grant from funding agencies in the public, commercial, or not‐for‐profit sectors.

## Disclosure

All authors read and approved the final manuscript.

## Ethics Statement

NHANES was approved by the Research Ethics Review Board of the National Center for Health Statistics, and all participants provided written informed consent. The present study is a secondary analysis of de‐identified public‐use data and therefore required no additional ethical approval.

## Consent

Please see the Ethics Statement.

## Conflicts of Interest

The authors declare no conflicts of interest.

## Supporting Information

Additional supporting information can be found online in the Supporting Information section.

## Supporting information


**Supporting Information** Supporting 1. Table S1. Baseline characteristics before (complete‐case) and after multiple imputation. Supporting 2. Table S2. Weighted quartile cutpoints of LAP and VAI. Supporting 3. Table S3. Variance inflation factors for Model 4 with mean‐centered LAP, VAI, and their product term. Supporting 4. Table S4. Supporting regression models: per 10‐unit increase, raw continuous scale and mean‐centered LAP × VAI interaction. Supporting 5. Table S5. Sensitivity analysis: complete‐case versus multiply imputed Model 3 estimates. Supporting 6. Figure S1. Sensitivity analysis: weighted restricted cubic spline curves for ln‐transformed LAP and ln‐transformed VAI with stroke, fitted on Model 3.

## Data Availability

NHANES is a publicly accessible dataset. All data used in this study can be downloaded free of charge from https://www.cdc.gov/nchs/nhanes/, and the analytic code is available from the corresponding author upon reasonable request.
