# Supplementary material for: Association Between Lipid Accumulation Product and Visceral Adiposity Index, as Markers of Visceral Adiposity and Metabolic Dysfunction, and Stroke: NHANES 1999–2018
Source: Int J Endocrinol. 2026 Oct 17;2026:6820633. doi: 10.1155/ije/6820633 (PMC13676159; doi:10.1155/ije/6820633)
Supplement: Supplementary file 1 [file IJE-2026-6820633-s001.zip › Supplementary tables.docx]

**Table S1. Baseline characteristics before (complete-case) and after multiple imputation.**

| **Characteristic** | **Pre-imputation (complete-case, n = 15,540)** | **Post-imputation (imputed set 1, n = 21,146)** | **P** |
| --- | --- | --- | --- |
| Age (years) | 49 (34, 64) | 49 (34, 64) | 0.057 |
| Male | 7,476 (48%) | 10,193 (48%) | 0.857 |
| Mexican American | 2,628 (17%) | 3,843 (18%) | 0.015 |
| Non-Hispanic White | 7,178 (46%) | 9,469 (45%) |  |
| Non-Hispanic Black | 2,978 (19%) | 4,057 (19%) |  |
| Married or living with partner | 9,583 (62%) | 13,153 (62%) | 0.438 |
| Less than high school | 3,808 (25%) | 5,598 (26%) | <0.001 |
| Below poverty line | 4,675 (30%) | 6,418 (30%) | 0.582 |
| Never smoke | 8,608 (55%) | 11,506 (54%) | 0.073 |
| Never drinking | 4,183 (27%) | 5,853 (28%) | 0.106 |
| Body mass index (kg/m2) | 28 (24, 32) | 28 (24, 32) | 0.015 |
| Waist circumference (cm) | 98 (88, 109) | 98 (88, 108) | 0.002 |
| Systolic blood pressure (mmHg) | 121 (111, 134) | 121 (111, 134) | 0.798 |
| HbA1c (%) | 5.50 (5.20, 5.80) | 5.50 (5.20, 5.80) | <0.001 |
| eGFR (mL/min/1.73m2) | 98 (81, 112) | 99 (82, 113) | <0.001 |
| Lipid accumulation product | 44 (25, 76) | 44 (24, 76) | 0.590 |
| Visceral adiposity index | 1.48 (0.90, 2.50) | 1.50 (0.91, 2.53) | 0.108 |
| Antihypertensive medication, yes | 5,488 (35%) | 7,066 (33%) | <0.001 |
| Lipid-lowering medication, yes | 3,004 (19%) | 3,748 (18%) | <0.001 |

Data are median (Q1, Q3) or n (%); unweighted. P values from Wilcoxon rank-sum and Pearson chi-square tests.

**Table S2. Weighted quartile cutpoints of LAP and VAI.**

| **Variable** | **Q1** | **Q2** | **Q3** | **Q4** |
| --- | --- | --- | --- | --- |
| Lipid accumulation product | < 23.072 | 23.072-42.272 | 42.272-74.855 | >= 74.855 |
| Visceral adiposity index | < 0.894 | 0.894-1.468 | 1.468-2.486 | >= 2.486 |

**Table S3. Variance inflation factors for Model 4 with mean-centred LAP, VAI and their product term.**

| **Variable** | **VIF** |
| --- | --- |
| LAP (centred) | 14.273 |
| VAI (centred) | 12.385 |
| LAP x VAI (centred) | 6.292 |
| Age | 2.885 |
| Sex | 1.486 |
| Race/Ethnicity | 1.349 |
| Marital status | 1.514 |
| Education level | 1.582 |
| Smoking status | 1.385 |
| Poverty-Income Ratio | 1.762 |
| Alcohol consumption | 1.372 |
| HbA1c | 1.671 |
| Systolic blood pressure | 1.418 |
| Antihypertensive medication | 1.956 |
| Lipid-lowering medication | 1.575 |
| eGFR | 2.399 |

Generalized VIF values for multi-category variables were rescaled as (GVIF^(1/(2xDf)))^2. VIF > 5 was taken to indicate serious collinearity.

**Table S4. Supplementary regression models: per 10-unit increase, raw continuous scale and mean-centred LAP x VAI interaction.**

| **Exposure** | **Model 1 OR (95% CI)** | **P** | **Model 2 OR (95% CI)** | **P** | **Model 3 OR (95% CI)** | **P** | **Model 4 OR (95% CI)** | **P** |
| --- | --- | --- | --- | --- | --- | --- | --- | --- |
| LAP (per 10 units) | 1.018 (1.010-1.026) | <0.001 | 1.019 (1.010-1.027) | <0.001 | 1.017 (1.009-1.024) | <0.001 | 1.006 (0.993-1.019) | 0.382 |
| LAP (raw continuous) | 1.002 (1.001-1.003) | <0.001 | 1.002 (1.001-1.003) | <0.001 | 1.002 (1.001-1.002) | <0.001 | 1.001 (0.999-1.002) | 0.382 |
| VAI (per 10 units) | 1.327 (1.136-1.551) | <0.001 | 1.402 (1.156-1.700) | <0.001 | 1.280 (1.072-1.528) | 0.007 | 1.084 (0.801-1.467) | 0.597 |
| VAI (raw continuous) | 1.029 (1.013-1.045) | <0.001 | 1.034 (1.015-1.054) | <0.001 | 1.025 (1.007-1.043) | 0.007 | 1.008 (0.978-1.039) | 0.597 |
| LAP x VAI (centred) | 1.000 (1.000-1.000) | 0.045 | 1.000 (1.000-1.000) | 0.014 | 1.000 (1.000-1.000) | 0.064 | 1.000 (1.000-1.000) | 0.938 |

Model definitions are as in Table 2. The interaction estimates are reported for completeness only; given severe collinearity (Table S3) and the scale of the product term, they are not interpretable.

**Table S5. Sensitivity analysis: complete-case versus multiply imputed Model 3 estimates.**

| **Exposure** | **Complete-case OR (95% CI)** | **P** | **Multiply imputed OR (95% CI)** | **P** |
| --- | --- | --- | --- | --- |
| LAP (per 1-SD) | 1.114 (1.045-1.188) | 0.001 | 1.115 (1.061-1.171) | <0.001 |
| VAI (per 1-SD) | 1.077 (1.003-1.156) | 0.043 | 1.073 (1.020-1.129) | 0.007 |
| LAP (per 10 units) | 1.017 (1.007-1.028) | 0.001 | 1.017 (1.009-1.024) | <0.001 |
| VAI (per 10 units) | 1.329 (1.012-1.745) | 0.043 | 1.280 (1.072-1.528) | 0.007 |
| LAP x VAI (centred) | 1.000 (1.000-1.000) | 0.281 | 1.000 (1.000-1.000) | 0.064 |

All estimates are from Model 3 with the combined fasting subsample weight.
